# DNA repair and disease: insights from the human DNA glycosylase NEIL family

**DOI:** 10.1038/s12276-025-01417-0

**Published:** 2025-03-03

**Authors:** Yuna Hwang, Su-Jin Kang, Jieun Kang, Jeongwoo Choi, Seung-Jin Kim, Sunbok Jang

**Affiliations:** 1 College of Pharmacy, Graduate School of Pharmaceutical Sciences, Ewha Womans University, Seoul, Republic of Korea; 2 Graduate Program in Innovative Biomaterials Convergence, Ewha Womans University, Seoul, Republic of Korea; 3 College of Pharmacy, Dongduk Women’s University, Seoul, Republic of Korea; 4 Department of Biochemistry, College of Natural Sciences, Kangwon National University, Chuncheon, Republic of Korea

**Keywords:** Protein-protein interaction networks, Base excision repair

## Abstract

The base excision repair pathway protects DNA from base damage via oxidation, deamination, alkylation and methylation. DNA glycosylases are key enzymes that recognize damaged bases in a lesion-specific manner and initiate the base excision repair process. Among these, the endonuclease VIII-like 1–3 (NEIL1–3) family, which is found in mammalian genomes, is a homolog of bacterial DNA glycosylases known as Fpg/Nei. NEIL enzymes have similar structures and substrates but with slight differences. When repair proteins are impaired, the accumulation of damaged bases can lead to increased genomic instability, which is implicated in various pathologies, including cancer and neurodegeneration. Notably, mutations in these proteins also influence a range of other diseases and inflammation. This review focuses on the influence of the NEIL family on human health across different organ systems. Investigating the relationship between NEIL mutations and diseases can improve our understanding of how these enzymes affect the human body. This information is crucial for understanding the basic mechanisms of DNA repair and enabling the development of novel inhibitors or gene therapies that target only these enzymes. Understanding the role of the NEIL family provides insights into novel therapies and improves our ability to combat genetic diseases.

## NEIL enzymes influence DNA repair and human health

The base excision repair pathway is crucial for fixing DNA damage, including oxidative base lesions, and maintaining genetic stability. This article reviews the role of NEIL enzymes, which are part of the base excision repair pathway, in various diseases. NEIL enzymes help to repair damaged DNA by removing faulty bases caused by oxidative or alkylation damage. The study highlights the diverse roles of NEIL1, NEIL2 and NEIL3 enzymes in various organ systems and diseases. The researchers found that NEIL1 is broadly associated with the nervous and cardiovascular systems, and uniquely affects the integumentary system and liver, while the functions of NEIL2 are primarily concentrated in the immune and respiratory systems and are specifically associated with bacterial infections of the stomach and large intestine. NEIL3 is linked to the cardiovascular and immune systems, but unlike other NEILs, it shows no independent organ-specific effects. This review also shows that these enzymes play roles in brain health, immune response and skin protection. These findings suggest that understanding NEIL enzymes can lead to better treatments for diseases related to DNA damage by enabling targeted therapies, such as small-molecule modulators, and guiding research to enhance DNA repair in specific diseases.

This summary was initially drafted using artificial intelligence, then revised and fact-checked by the author.

## Introduction

### BER pathway

The base excision repair (BER) pathway effectively repairs damaged DNA and maintains genome integrity^1–3^. Numerous diseases, including neurodegenerative disorders and age-related conditions, are associated with BER enzyme defects. This complicated system protects genetic information from damage and ensures faithful DNA replication and gene expression.

**Fig. 1:**
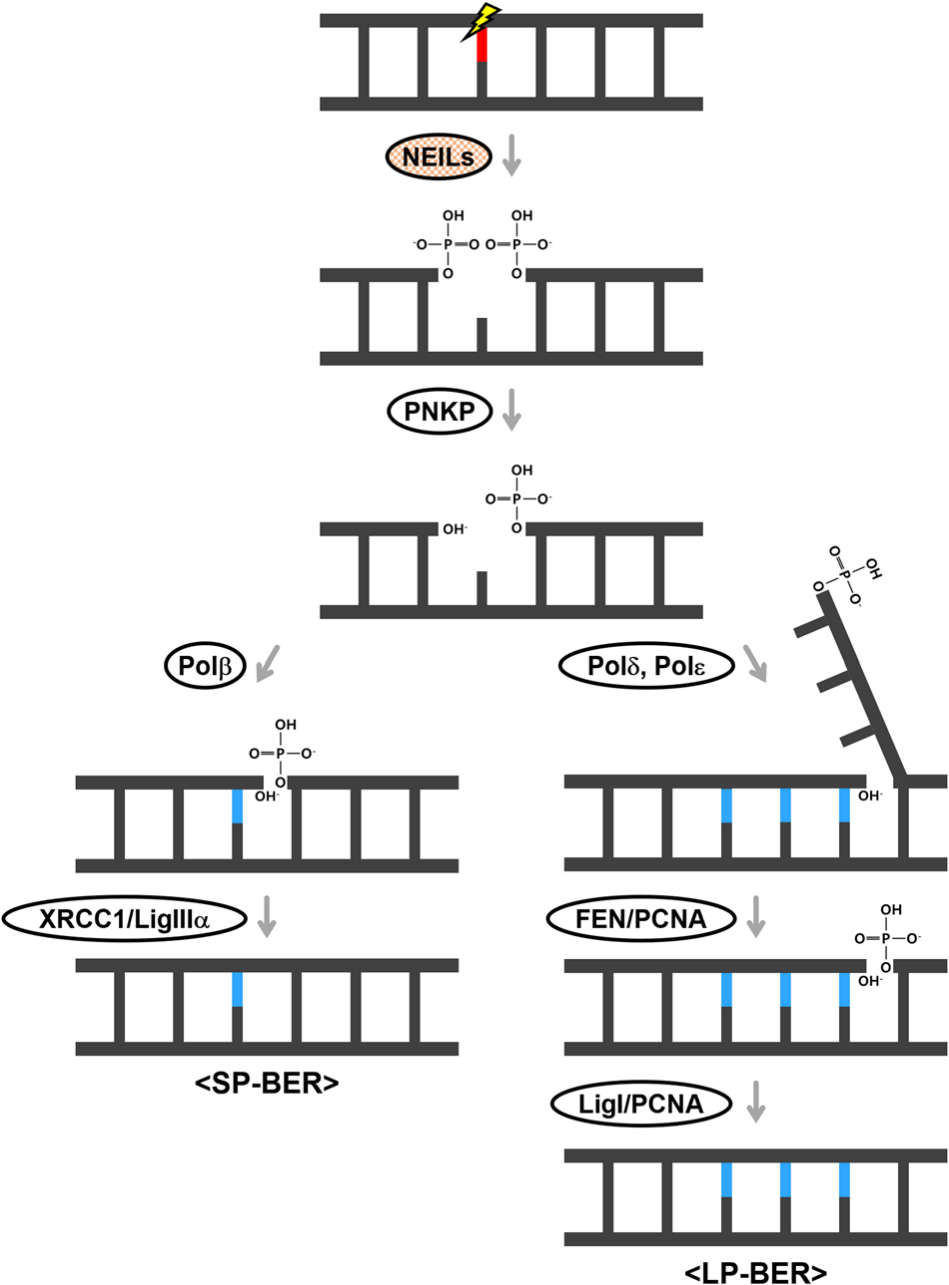
BER pathway focusing on NEILs. When damage occurs at a damaged base (red), the BER pathway is initiated by DNA glycosylases, including NEILs, leading to the repair of the base (blue).

As shown in Fig. 1, the BER pathway begins with DNA glycosylases that detect damaged bases^3,4^. DNA glycosylases can be classified into monofunctional glycosylases and bifunctional glycosylases^5,6^. Among bifunctional glycosylases, our focus is on the endonuclease VIII-like family (NEILs), which directly cleave the DNA backbone, creating a single-stranded nick. The bifunctional glycosylases include 8-oxoguanine glycosylase, endonuclease III-like protein 1 (NTH1) and NEIL1–3 (refs. ^7,8^).

After bifunctional glycosylases excise the DNA backbone, polynucleotide kinase 3′-phosphatase (PNKP) converts the terminal 3′-phosphor-α,β-unsaturated aldehyde (3′-PUA) or 3′-phosphate of the cleaved strand into OH groups^9^. In the next step, BER pathways are classified according to the enzyme types involved in the repair patch region. In short-patch BER (SP-BER), DNA polymerase β (polβ) attaches nucleotides to the OH-terminal DNA backbone, and XRCC1/LigIIIα seals the nick^10,11^. In long-patch BER (LP-BER), DNA polymerase δ or ε (polδ or polε) synthesizes the damaged base and extends synthesis to the area following it^12,13^. The flap structure-specific endonuclease (FEN)/proliferating cell nuclear antigen (PCNA) complex removes the original DNA backbone, and the LigI/PCNA complex seals the nicks^1,14–17^.

### NEIL family

The endonuclease III (NTH) family was discovered in 1976^18^, and its homolog, the *Nei* gene, was subsequently identified in *Escherichia coli* in 1994^19^. Mammalian counterparts of the Fpg/Nei family of *E.* *coli* are known as Nei-like glycosylases and share conserved helix-2-turn-helix (H2tH) motifs with *E.* *coli* Fpg/Nei proteins. In the early twenty-first century, the Wallace, Mitra and Seeberg laboratories identified mammalian homologs of Fpg/Nei DNA glycosylases, such as NEIL1, NEIL2 and NEIL3 (refs. ^20–24^). NEIL1 and NEIL2 were isolated and purified relatively early, allowing a more detailed understanding of their biochemical properties^20–25^. In this review, we specifically use the term ‘NEILs’ to refer to human enzymes, whereas ‘Neils’ is used for mouse enzymes.

As presented in Table 1, the proteins in the NEIL family have some similarities and differences in terms of their structural conformations and the substrates on which they act. The NEIL family exhibits differences in substrate specificity compared with DNA glycosylases. NEIL1 and NEIL2 bind well to ring-fragmented or saturated pyrimidines, including thymine glycol (Tg), 2,6-diamino-4-hydroxy-5-formamidopyrimidine (FapyG), 5-hydroxycytosine (5-hC), 5-hydroxyuracil (5-OHU) and 4,6-diamino-5-formamidopyrimidine (FapyA)^21,23,25–28^. Compared with unedited NEIL1, edited NEIL1 exhibits greater catalytic efficiency for FapyA and FapyG. However, its activity is lower on an oligodeoxynucleotide containing the aflatoxin B1 (AFB1)–FapyG adduct^29^. Although NEIL3 is less studied than NEIL1 and NEIL2, it also binds to FapyG, FapyA and Tg^30^. Studies have demonstrated that human NEIL3 exhibits substrate specificity, acting on single-stranded and double-stranded DNA^30,31^. Notably, all NEIL family members, such as guanidinohydantoin (Gh) and spiroiminodihydantoin (Sp), have high affinity for imidazole ring-opened DNA lesions^31–33^.

**Table 1:** Characteristics of NEIL1, 2 and 3.

Proteins	Preferred substrates	Primarily expressed cell cycle phase	Structure details	Refs.
NEIL1	FapyG, FapyA, Tg, 5-hC, 5-hU, Sp, Gh (ring fragmented, saturated pyrimidine, hydantoin)	S phase (replication)	Similar to Fpg/Nei in the N-terminal region with an H2tH motif resulting in an αG helix	^1–35,38,39^
NEIL2	FapyG, FapyA, Tg, 5-hC, 5-hU, Sp, Gh (ring fragmented, saturated pyrimidine, hydantoin)	Continuous (transcription)	Similar to Fpg/Nei in the N-terminal region with an H2tH motif resulting in an αG helix; C-terminal zinc finger motif	^1–33,39^
NEIL3	FapyG, FapyA, Sp, Gh	S, G2 phase	Similar to Fpg/Nei in the N-terminal region with an H2tH motif resulting in an αG helix; elongated C-terminal domain	^29–33,37–39^

NEIL family proteins have commonalities and differences in their preferred substrates and structures. Additionally, the primary phases of the cell cycle are slightly different.

Hedge et al. revealed that NEIL1 interacts with various replication proteins, including FEN-1, PCNA and replication protein A, and its expression is regulated by the S phase of the cell cycle. The authors reported that NEIL1 plays a crucial role in replication and removing lesions such as 5-OHU, which can cause mutations^34,35^. In contrast, NEIL2 exhibits an affinity for lesions in single-stranded DNA and bubble structures. NEIL2 is involved in transcription-coupled repair processes through interactions with transcription factors, including RNA polymerase II^22,24,25,36^. The expression of human NEIL3 varies throughout the cell cycle, reaching peak levels during the G2 phase, although expression also occurs during the S phase^37,38^.

**Fig. 2:**
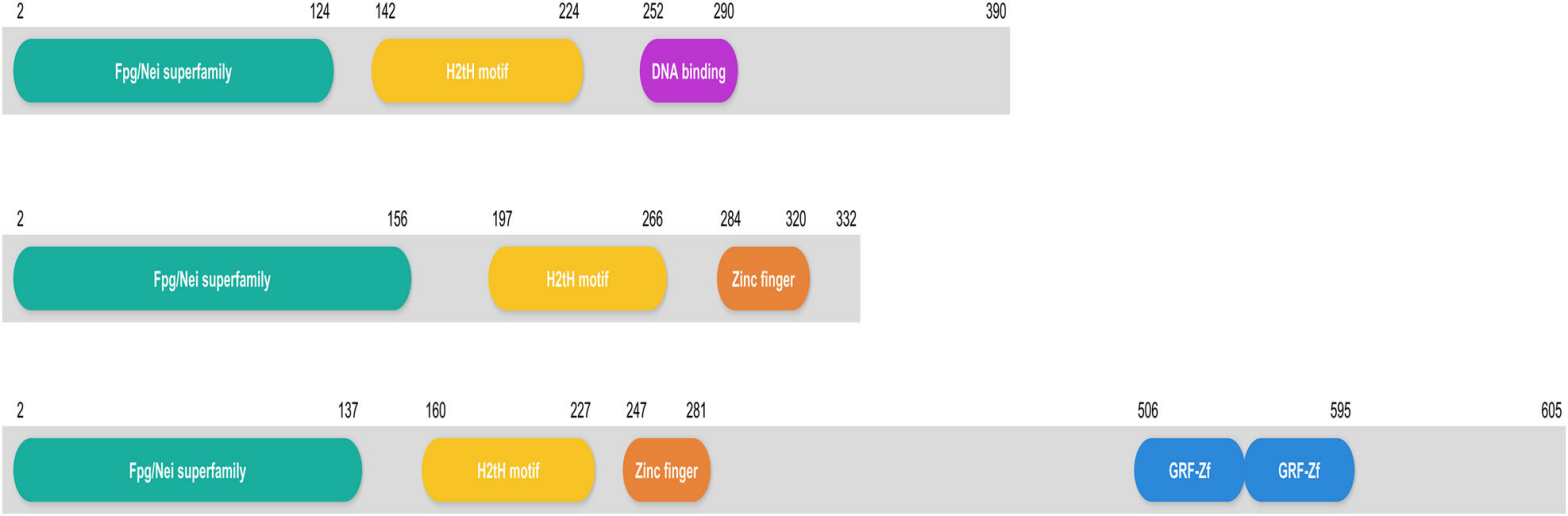
NEIL protein domains. NEIL1 contains three domains: the Fpg/Nei superfamily (green), the H2tH motif (yellow) and DNA-binding domains (purple). NEIL2 consists of three domains: the Fpg/Nei superfamily (green), the H2tH motif (yellow) and the zinc finger (orange). NEIL3 has four different domains: the Fpg/Nei superfamily (green), H2tH motif (yellow), zinc finger (orange) and two glycine–arginine–phenylalanine (GRF)-type zinc finger (GRF-Zf) domains (blue). Each of these domains is positioned at specific locations along the amino acid sequence, helping these proteins perform their DNA repair functions.

In general, NEIL proteins are similar to Fpg/Nei proteins in their N-terminal region, which contains the H2tH motif resulting in an αG helix. NEIL2 has a C-terminal zinc finger motif and shares large similarities with Fpg and Nei. However, NEIL3 has an elongated C-terminal domain that extends beyond the conserved Fpg/Nei sequence. NEIL3 has a zinc finger motif containing a secondary C-terminal motif related to the RanBP zinc finger family, similar to that observed in Fpg/Nei^39^. Figure 2 clearly depicts the domain-specific characteristics of NEIL proteins.

### Diseases related to the NEIL family

Recent research has demonstrated that the NEIL family is notably involved in the development and progression of various cancers. Research from diverse perspectives, including in vivo and in silico work, has revealed the involvement of NEILs in the DNA repair process of cancer cells. Many researchers have comprehensively reviewed the relationship between the NEIL family and cancers. In particular, many ongoing investigations are examining the potential of NEILs in contributing to cancer diagnosis, prevention and treatment^40–42^.

Many cancers are caused by various inflammatory responses^43,44^. Inflammation is an important precursor to cancer development, and early inflammatory responses can promote the formation and growth of cancer cells. Therefore, understanding how the NEIL family interacts with infections is crucial for cancer research and the study of inflammatory diseases. Moreover, almost a year has passed since the emergence of the coronavirus disease 2019 (COVID-19) pandemic. COVID-19 has reminded us of the unexpected ripple effects of infectious diseases.

Reactive oxygen species (ROS) refer to include oxygen-derived radicals such as superoxide (O₂⁻), hydrogen peroxide (H₂O₂), and hydroxyl radicals (•OH). They are produced during inflammation to prevent infections and promote tissue regeneration and repair, they may also damage DNA, which can lead to mutations that cause cancer^45^. Therefore, understanding the role of the NEIL family is crucial, especially in diseases related to epidemics, inflammation and infection.

We reviewed research related to the NEIL family, focusing on the associations of this enzyme group with inflammatory responses, infectious diseases and other diseases beyond their known roles as DNA glycosylases to provide an in-depth analysis of their influence on the development and progression of inflammatory and infectious diseases. Our review contributes to the development of future diagnostic and treatment strategies and enables researchers to explore new perspectives and approaches in cancer, inflammation and infectious disease research.

## Digestive system

Infections with AFB1 are associated with hepatitis B virus (HBV) infection, hepatitis C virus (HCV) infection and cancer development, including hepatocellular carcinoma (HCC). Epidemiological data demonstrate that HCC risk is significantly influenced by dietary intake of AFB1 and persistent HBV infection^46,47^. NEIL1 has been demonstrated to inhibit AFB infections. NEIL1 efficiently identifies and removes the highly mutagenic imidazole ring-opened AFB1–deoxyguanosine adduct (AFB1–Fapy-dG). Reports have demonstrated that NEIL1 can catalyze the repair of most AFB1–Fapy-dG adducts by initiating BER. Patients with NEIL1 variants appear to experience problems related to protein stabilization, protein localization, domains responsible for protein‒protein interactions and genetic transcription/translation processes^48^. These problems contribute to an increase in the steady-state levels of AFB–Fapy-dG adducts.

Some studies have reported that exposure to AFB can induce the progression of certain NEIL1 mutations to HCC. Zuckerman et al.^49^ suggested a potential association between naturally occurring single-nucleotide polymorphism (SNP) variations in NEIL1 and increased susceptibility to early onset HCC. This correlation is particularly pronounced in settings with high aflatoxin exposure^49^. This research provides valuable insights into the potential increased risk of early onset HCC in populations carrying the NEIL1 I182M variant. The NEIL1 A51V variant also increases the risk of HCC by reducing the rate of damage excision and temperature sensitivity^50^.

Compared with uninfected cells, human HCC cells infected with HCV JFH-1 exhibit 30–60-fold increased ROS levels. Notably, NEIL1 expression levels are significantly decreased in these HCV-infected cells (unlike other glycosylases). Liver biopsies from patients with advanced HCV infection reveal remarkably lower NEIL1 levels than those from patients without disease^51^.

Moreover, *Helicobacter pylori*, a bacterium with the potential to induce gastric cancer, is also inhibited by NEIL2. *H.* *pylori* disrupts the BER pathway, which fixes oxidized DNA lesions, and produces ROS by vacuolating factors such as CagA and VacA^52^. This disruption leads to the accumulation of oxidatively damaged bases, increasing the risk of mutations and genomic instability. The persistent inflammation, ROS production and DNA damage caused by *H.* *pylori* infection may contribute to the progression of gastric cancer^53,54^. Reports have shown that decreased NEIL2 expression levels facilitate the accumulation of DNA damage secondary to *H.* *pylori*^55^. *H.* *pylori* infection reduces NEIL2 expression in a manner dependent on the duration and intensity of infection. This reduction in NEIL2 promotes the accumulation of DNA damage, which may play a key role in the development of gastric cancer^55^. Studies have further demonstrated a correlation between decreased NEIL2 levels in human gastric tissue and the progression of gastric cancer. By inhibiting NEIL2, *H.* *pylori* hinders DNA damage repair mechanisms, amplifies inflammation and facilitates gastric cancer development^55^.

NEIL2 is also involved in colorectal cancer (CRC) progression caused by *Fusobacterium nucleatum* (Fn) infection. Fn reduces the expression of the BER protein NEIL2 in a murine CRC model^56^. Fn infection of human enteroid-derived monolayers from Neil2-null mice resulted in severe DNA damage, including DNA double-strand breaks and inflammatory cytokines. Transcriptomic analysis revealed that Neil2 downregulation was more common in microsatellite stable CRC than in microsatellite instability CRC. Fn induces NEIL2 downregulation, resulting in the accumulation of DNA damage and contributing to CRC progression^57^.

Primary sclerosing cholangitis (PSC) is a chronic inflammatory disease that induces bile duct stricture and liver sclerosis^58^. Considering the significant risk of cholangiocarcinoma (CCA) due to the malignant transformation of biliary epithelial cells in PSC, recent studies have revealed a 6–13% incidence rate for CCA in patients with PSC^59–61^. Genetic variants may influence the risk of progression from PSC to CCA. Studies have shown that three rare variants, including NEIL1 G83D, identified in patients with PSC and PSC–CCA can substantially affect protein function^62^. In tissues such as bile ducts with severe oxidative stress in PSC patients, decreased DNA repair leads to increased levels of oxidative DNA damage, resulting in increased rates of genetic mutations.

## Respiratory system

Severe acute respiratory syndrome coronavirus 2 (SARS-CoV-2) infection increases the expression of soluble inflammatory mediators, leading to the influx of inflammatory cells, such as macrophages, T cells and neutrophils, to the infection site^63,64^. This uncontrolled inflammation leads to pulmonary endothelial leakage and impairs lung function^65^. CoV-2 infection and host inflammatory responses generate ROS, which damage the host genome and trigger a DNA damage response^66^.

The ability of individuals to repair damaged DNA may notably influence the severity of SARS-CoV-2 infection-induced COVID-19. NEIL2 specifically binds to the 5′-untranslated region (5′-UTR) of SARS-CoV-2 genomic RNA and inhibits protein synthesis. These findings suggest that NEIL2 plays a novel role in regulating CoV-2-induced pathogenesis that was previously unidentified. NEIL2 specifically binds to the 5′-UTR of SARS-CoV-2 genomic RNA, inhibiting viral replication and preventing inflammatory responses^67^. Analysis of publicly available transcriptomic datasets from patients with COVID-19 revealed significantly lower expression of NEIL2 in the lungs of patients with severe symptoms^68^.

Radiation pneumonitis (RP) is a common side effect in patients with lung cancer receiving radiotherapy and represents an acute clinical symptom of radiation-induced lung injury^69^. RP can have fatal outcomes in some cases, with severe RP having a mortality rate of up to 50% (ref. ^70^). Genetic analysis of *NEIL1* gene polymorphisms in patients with lung cancer undergoing radiotherapy revealed an association between the rs7402844 GG genotype and a greater risk of RP grade ≥2 than the rs4462560 CC genotype. Thus, *NEIL1*-mutant patients with lung cancer have a greater risk of RP than other patients do^71^.

Deficiencies of Neil1 and Nth1 lead to a greater frequency of lung and liver tumors in mice^72^. The absence of Neil1 in the repair of oxidatively damaged DNA leads to the accumulation of carcinogenic oxidative DNA damage, which is not limited to 8-oxoG, contributing to tumor and cancer development in the lung and liver.

## Cardiovascular system

NEIL1 deficiency can lead to metabolic syndrome, obesity and liver inflammation. Exposure to chronic oxidative stress in the form of a high-fat diet considerably accelerates obesity onset in Neil1^−/−^ mice. Moreover, high-fat diet-fed Neil1^−/−^ mice exhibit significantly greater expression of inflammatory genes in the liver than do chow-fed or Neil1^+/+^ mice. The absence of Neil1 potentially lowers the threshold for resistance to cellular oxidative stress in Neil1^−/−^ mice, increasing their susceptibility to obesity and its associated complications^73^. Moreover, Neil1^−/−^ and Neil1^+/−^ mice exhibit morbid conditions, such as obesity, dyslipidemia, fatty liver disease and hyperinsulinemia, even in the absence of such external oxidative stress^74^. Together, these conditions are known as metabolic syndrome in humans. Furthermore, analysis of Neil1^−/−^ mice mitochondrial DNA revealed greater levels of ROS-induced DNA damage and loss than those in wild-type (WT) mice, highlighting that Neil1 plays a crucial role in preventing diseases associated with metabolic syndrome.

Myocardial infarction (MI) induces a reparative response involving the fibroblast proliferation and differentiation required to form scar tissue for stabilization^75^. Recently, genetic variants in NEIL3 have been associated with an increased risk of MI in humans. One study investigated the relationship between the NEIL3 SNP and the development of MI^76^. The results revealed that individuals carrying the NEIL3 rs12645561 TT genotype exhibited a notably greater risk of MI than did those with the rs12645561 CC or rs12645561 CT genotypes. These findings suggest a remarkable association between the NEIL3 SNP and susceptibility to MI. Therefore, the NEIL3 SNP may play a role in atherogenesis development.

Neil3^−/−^ mice exhibit increased proliferation of fibroblasts and myofibroblasts after MI, which may lead to cardiac rupture and increased mortality. Genome-wide analysis data related to 5-methylcytosine and 5-hydroxymethylcytosine suggest that Neil3-dependent regulation of DNA methylation regulates cardiac fibroblast proliferation and extracellular matrix formation^77^.

Furthermore, oxidative stress causes DNA damage both in the nucleus and in the mitochondria, and reports have demonstrated that DNA damage accumulates in atherosclerotic lesions^78^. Therefore, the DNA repair system may impact the development of atherosclerosis^79^. NEIL3 not only hydrolyzes glycosidic bonds but also affects lipid metabolism, contributing to the prevention of atherosclerosis. NEIL3 controls lipid metabolism in the liver and affects cholesterol movement in macrophages. Its absence leads to accelerated atherosclerosis by changing vascular smooth muscle cell (VSMC) behavior and disrupting lipid balance in Apoe^−/−^ mice^80,81^.

Bente Halvorsen and colleagues performed various studies comparing apolipoprotein E (Apoe)^−/−^ Neil3^−/−^ mice with Apoe^−/−^ mice^80^. Apoe^−/−^ Neil3^−/−^ mice fed a high-fat diet presented accelerated plaque formation and substantial impairments in various pathways affecting hepatic lipid metabolism compared with Apoe^−/−^ mice. However, there were no significant changes in genome stability or accumulation of oxidative DNA damage. These findings suggest that NEIL3 plays a role in regulating lipid metabolism and macrophage balance and is unrelated to its function as a DNA glycosylase, thus inhibiting atherosclerosis. A 2021 study revealed the mechanisms through which NEIL3 affects atherosclerosis^81^. VSMCs are significantly affected by atherosclerosis^82,83^. Research using Apoe^−/−^ Neil3^−/−^ mice and NEIL3-deficient human VSMCs revealed that NEIL3 deficiency induces a change in the VSMC phenotype without altering DNA damage. This phenomenon is associated with increased activation of the Akt signaling pathway, which promotes atherosclerosis through Akt-dependent proliferation in NEIL3-deficient VSMCs^81^.

## Nervous system

NEIL1 deficiency causes neuroinflammation, which can lead to Parkinson’s disease (PD), stroke and defects in the development of cranial neural crest cells. The accumulation of DNA damage due to ionizing radiation (IR) increases the risk of neurodegenerative diseases in NEIL1-deficient individuals^84^. In open field tests, Neil1-deficient mice exhibited anxiety-related behaviors and cognitive memory deficits in novel object recognition and increased neuroinflammatory responses under baseline conditions. Compared with WT mice, these mice exhibited reduced neurogenesis due to IR-induced stress, a lack of neuroinflammation resolution and increased DNA damage and apoptosis in the hippocampus.

PD is a movement disorder caused by the loss of dopaminergic neurons and the degeneration of dopaminergic terminals in the striatum^85–87^. NEIL1 plays a crucial role in limiting the degeneration of dopaminergic neurons due to oxidative stress. Compared with WT mice, Neil1^−/−^ mice treated with 1-methyl-4-phenyl-1,2,3,6-tetrahydropyridine exhibited greater motor dysfunction, nigrostriatal pathway degeneration and DNA damage accumulation^88^. This finding suggests that NEIL1 is an important defensive molecule in oxidative cellular environments^88,89^.

Stroke is a medical condition characterized by damage to brain tissue caused by a blockage or hemorrhage in a blood vessel in the brain, disrupting the blood supply to the brain. Risk factors include high blood pressure, diabetes, atherosclerosis, elevated homocysteine levels and genetic predispositions. This condition causes rapid cell death in affected brain regions and is a leading cause of disability in adults worldwide^90,91^. NEIL1, as a DNA glycosylase, also protects neurons from memory impairments and other damage caused by stroke^92^. A study on memory retention in Neil1-deficient mice used a water maze test and explored the protective role of Neil1 against ischemic injury in a focal ischemia/reperfusion stroke model. Researchers have measured the incision capacity of a 5-hU-containing bubble substrate in ischemic brain regions and mitochondrial lysates to assess DNA repair efficiency. This study highlights the key role of Neil1 in neuronal protection and memory, suggesting its potential as a therapeutic target for stroke and neurodegeneration.

NEIL1 and NEIL2 function as BER enzymes and are involved in DNA demethylation, suggesting their involvement in development and disease. Research has revealed that the absence of Neil2 in frogs leads to facial and cranial deformities^93^. Studies on frog embryos and mouse embryonic stem cells have demonstrated that these cells lose the ability to develop into cranial neural crest cells without Neil1 and Neil2. Typically, Neil1 and Neil2 in the nucleus do not act as DNA repair enzymes during the differentiation of stem cells into cranial neural crest cells. However, mitochondrial Neil1 and Neil2 are involved in DNA damage repair during this period. NEIL deficiency causes specific oxidative damage to mitochondrial DNA, leading to TP53-mediated endogenous cell death. Therefore, NEIL1 and NEIL2 DNA glycosylases protect mitochondrial DNA from oxidative damage during neural crest differentiation^94^.

Keratoconus (KC) is a progressive corneal disease that leads to vision loss and is characterized by thinning and protrusion of the cornea^95^. Changes in corneal curvature can result in myopia and irregular astigmatism^96^. While the exact mechanisms of KC onset remain unclear, reports have linked oxidative stress to this condition^97–99^. A study estimating the frequency of five SNPs in the BER gene in the Polish population suggested a possible association between KC and SNPs in NEIL1 (ref. ^99^).

Age-related cataracts (ARCs) can lead to vision loss due to lens opacification, and various factors, including genetics, ultraviolet (UV) light exposure and diabetes, influence cataract development. Epidemiological studies have revealed that different types of cataracts are caused by a combination of lifestyle and environmental factors, such as smoking, drinking and higher education levels, which influence the development and progression of cataracts. Genotyping analysis within the microRNA (miRNA) region of NEIL2 for SNPs and its association with ARC sensitivity revealed a significant correlation with rs4639:T > C. Post-transcriptional gene regulation by miRNA-mediated SNP changes could be a potential pathogenic mechanism for ARCs. SNPs affecting miRNA binding to the 3′-UTR of BER pathway genes may contribute to variable disease susceptibilities^100^.

Prion diseases are fatal neurodegenerative conditions caused by misfolded proteins, leading to brain damage with no cure. These include disorders, such as Creutzfeldt–Jakob disease, and potentially contribute to Alzheimer’s disease (AD) and PD. These diseases involve toxic protein aggregates that generate oxidative stress, leading to the accumulation of oxidative DNA damage^101–103^. Studies have reported that NEIL2 and NEIL3 employ distinct mechanisms for DNA protection; NEIL2 mediates the acute response against toxic signals, whereas NEIL3 functions in neurodevelopmental protection^104,105^. Researchers have infected Neil2^−/−^ mice with prions and examined disease progression in the brain and spleen, along with DNA damage accumulation and mitochondrial respiratory complex activity. The results revealed that Neil2^−/−^ mice deteriorated more rapidly. Neil2 responds to toxic signals in clinical prion diseases^105^. When Neil3^−/−^ mice were infected, the incubation period was similar to that of WT mice; however, the duration of infection was shorter. NEIL3 protects against prion disease exacerbation due to oxidative DNA damage during neurodevelopment^104^.

The brain is primarily composed of easily oxidized lipids^106^. The brain exhibits high oxygen consumption, which indicates that DNA damage-generating oxidative stress is a key feature of AD^107^. The DNA glycosylase function of NEIL3 in the brain is associated with hippocampus-dependent memory and neurogenesis after stroke and in prion diseases. In a new AD mouse model lacking Neil3, female Neil3-deficient mice exhibited decreased amyloid-β plaque deposition with age, whereas male Neil3-deficient AD mice exhibited reduced neural stem cell proliferation in the adult hippocampus and impaired working memory compared with controls. This finding indicates the involvement of Neil3 in regulating cerebral amyloid-β accumulation and promoting adult hippocampal neurogenesis to maintain cognitive functions during AD progression^108^.

Astrocytoma is the most common type of primary brain tumor and is known for its high degree of treatment resistance and rapid disease progression^109,110^. Research has revealed the impact of DNA repair genes, especially NEIL3, on glioblastoma progression and resistance. NEIL3 knockdown increased DNA damage (as measured by γ-H2A X staining) and cell apoptosis following irradiation of glioblastoma cells, suggesting enhanced efficiency in the repair of double-strand breaks and resistance to reactive oxygen species^111^.

## Immune system

The nuclear factor kappa-light-chain-enhancer of activated B cells (NF-κB) is a key transcription factor complex that regulates gene expression, cytokine production and cell survival. NF-κB responds to various stimuli, including stress and pathogens, across almost all animal cell types. This system is crucial to cellular defense and regulatory mechanisms^112–115^. NEIL2 is particularly associated with NF-κB-mediated inflammation. Studies have shown that NEIL2 inhibits the expression of NF-κB-mediated proinflammatory genes by blocking their binding to the promoters of proinflammatory genes. The results indicate a significant reduction in inflammatory responses in Neil2^−/−^ mice and WT animals upon intranasal administration of purified NEIL2, supporting these findings^116^.

Thus, NEIL2 actively recognizes oxidative DNA damage induced by respiratory syncytial virus (RSV) infection, setting the threshold for type I interferon-β (IFN-β) expression. NEIL2 counteracts the action of NF-κB on the IFN-β promoter shortly after infection, thus restraining the amplification of gene expression by IFNs. Mice lacking Neil2 are significantly more susceptible to RSV-induced illness, demonstrating elevated expression of proinflammatory genes and tissue damage^117^. However, these deficiencies were reversed by administering the Neil2 protein to the airways of the mice. These results underscore the protective role of NEIL2 in regulating IFN-β levels during RSV infection. Given the potential adverse effects of the use of type I IFNs in antiviral therapy, both short term and long term, NEIL2 could provide an alternative approach. NEIL2 ensures genome stability and contributes to controlling the immune response^117^.

Apoptotic cell clearance begins with pathogen recognition and engulfment by phagocytes^118^. An imbalance in cell death and debris clearance exposes self-antigens, leading to autoimmunity^119^. A NEIL3 variant causing a loss of function has been linked to systemic lupus erythematosus due to defective DNA clearance^120^. NEIL3 deficiency is associated with increased lymphocyte apoptosis, autoantibody production and a predisposition toward autoimmunity. Neil3^−/−^ mice exhibit increased apoptosis of splenic T and B cells, indicating an increased risk for autoimmunity due to the release of self-antigens. Furthermore, a homozygous missense mutation in NEIL3 was identified in three siblings, resulting in a loss of enzymatic activity and a potential for autoimmunity^121^.

## Integumentary system

NEIL proteins are also related to the integumentary system, which is an anatomic term that includes all components of body coverage, such as the skin, hair and nails^122^. Chronic UV radiation exposure increases ROS production, thus activating cellular signaling and proinflammatory cytokines^123^. Moreover, UV radiation downregulates sirtuin 1 expression in human keratinocytes, leading to chronic inflammation^124,125^.

Neil1^−/−^ mice also exhibit high levels of chronic inflammation when exposed to chronic UV radiation, suggesting that NEIL1 is also associated with UV-induced inflammation. Calkins et al. evaluated the extent of persistent DNA damage caused by ROS in the skin of mice chronically exposed to UVB to elucidate the state of chronic skin inflammation^126^. An elevated level of ROS-induced DNA lesions in the normal state was measured after chronic UVB exposure for the first time. Three oxidative lesions, FapyA, FapyG and Tg, were increased in the skin of mice chronically exposed to UVB. The heightened normal state suggests a key role of these lesions in the ROS-induced mutations observed in UV carcinogenesis^122^.

## Conclusions

There is limited documentation of the impact of NEILs on inflammation or diseases other than cancer. However, understanding the associations with other diseases beyond cancer is helpful for understanding NEILs and the BER pathway.

**Fig. 3:**
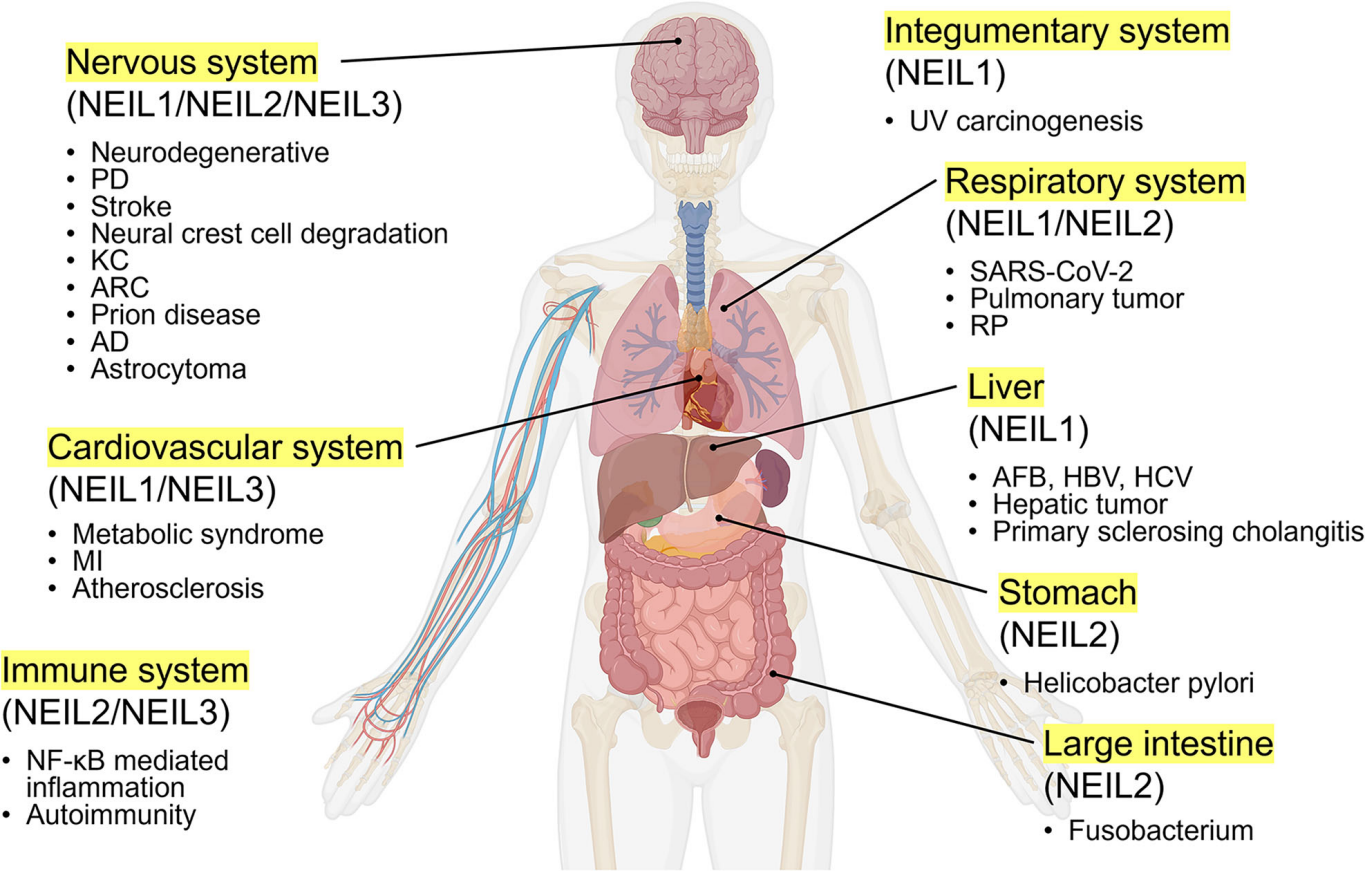
NEILs associated with inflammation and other diseases. NEIL1–3-related diseases are categorized into six body systems: the nervous, cardiovascular, immune, integumentary, respiratory and digestive systems. This classification highlights the diverse roles of NEIL family members in human health and disease. Figure created with BioRender.com.

As shown in Fig. 3, we categorized the effects of NEILs on various organs. Overall, NEIL1 and NEIL2 have been the focus of most disease-related research, a trend attributed to the later discovery of NEIL3. All NEILs affect the nervous system^84,88,89,92–94,99,100,104,105,108,111^. NEIL1 is broadly associated with the nervous and cardiovascular systems and uniquely linked to the integumentary system and liver^48–51,73,74,84,88,89,92,94,99,126^. NEIL2 functions are concentrated in the immune and respiratory systems, with its association exclusively noted in bacterial infections in the stomach and large intestine^67,68,116,117^. NEIL3 is reported to affect the cardiovascular and immune systems^80,81,120,121^. However, there is no known evidence that NEIL3 independently affects any organ, which is distinct from other NEILs.

Studies on the NEIL family and its associations with various diseases, including gene therapies and inhibitors, can enable drug development. DNA damage repair is critical for maintaining the integrity of genetic information and preventing the onset of numerous diseases^1–3^. By understanding how each NEIL protein is linked to specific diseases, researchers can develop new treatments that regulate the activity of these proteins. For example, increasing NEIL expression levels could be beneficial in situations with excessive DNA damage. In cancers with abnormal repair processes, NEIL inhibitors can be good targets for therapy.

Understanding the NEIL family will also help identify potential biomarkers for diseases caused by DNA damage, enable early disease detection and accelerate treatment. For example, by identifying specific genetic signatures associated with DNA repair mechanisms, doctors can customize treatment strategies on the basis of each patient’s unique genetic profile, thereby increasing the effectiveness of therapeutic measures. This precision medicine approach improves patient outcomes and minimizes the risk of side effects, leading to more effective and patient-focused health care.
